# Assessing veterinary students’ attitudes towards pain in cattle before and after final‐year clinical training

**DOI:** 10.1002/vetr.70731

**Published:** 2026-05-13

**Authors:** Theresa Tschoner, Kristina R. Mueller, Yury Zablotski, Melanie Feist

**Affiliations:** ^1^ Clinic for Ruminants with Ambulatory and Herd Health Services Centre for Clinical Veterinary Medicine Ludwig Maximilians University Munich Oberschleissheim Germany; ^2^ School of Veterinary Science Massey University Palmerston North New Zealand

**Keywords:** analgesics, pain assessment, pain management, ruminants, survey

## Abstract

**Background:**

Cattle only show subtle signs of pain, making pain assessment difficult. The approach to pain and the use of analgesics depend on the treating person's experience and opinions.

**Methods:**

Bavarian veterinary students were asked to answer two identical questionnaires before and after 12 weeks of clinical training in a ruminant clinic. A total of 60 students answered both questionnaires.

**Results:**

Out of 28 behavioural expressions of pain, six parameters were selected significantly more often following clinical training. Following training, pain scoring changed significantly for 10 of 20 conditions and three of 15 procedures in adult cattle, with enucleation of the eye bulb, claw amputation and caesarean section judged to be the most painful procedures. Castration (both surgical and Burdizzo) and laparotomy were scored as the most painful procedures in calves. Selected analgesic regimens did not change significantly, except for management for tenotomy of contracted tendons in calves. Students’ opinions about farmers’ attitudes towards pain and willingness to pay for analgesics differed after clinical training.

**Limitations:**

The high proportion of female participants could have influenced the study's results.

**Conclusion:**

Practical experience influences veterinary students’ attitudes towards pain recognition in cattle, but not towards pain management. Working with farmers changes students’ opinions about farmers’ preferences.

## INTRODUCTION

According to veterinary association oaths, veterinarians have a professional duty to relieve pain and suffering in animals.^1^ Pain in animals, whether caused by disease, husbandry practices or veterinary procedures, is a frequent welfare concern.^2^, ^3^, ^4^, ^5^ Its identification, assessment and quantification are important for effective treatment.^6^ Pain management in cattle is fundamental to prevent and manage pain in animals,^3^ and veterinarians play a central role in ensuring cattle welfare.^4^ Cattle are not able to self‐report pain, and the assessment of pain by veterinarians will always be subjective to a certain degree.^5^ People respond differently to their own pain, resulting in a wide range of veterinarians’ attitudes towards pain in animals^7^ and individual differences in pain evaluation.^5^

Cattle, as prey animals, strongly mask their pain, making the evaluation and assessment of pain difficult,^4^, ^7^, ^8^ often leading to the assumption that cattle are insensitive to pain.^8^ In addition to the limited ability to identify and assess pain in cattle, various other factors, such as inadequate analgesic interventions, research deficiencies (limited use of farm animals in pain research or lack of market demand for analgesic drugs in farm animals), and public expectation and perception (the issue of pain being overshadowed by topics such as movement restriction and space allowance), have led to limited efforts to alleviate pain in farm animals.^6^

Administration of analgesics in cattle depends on veterinarians and producers recognising pain.^2^ However, even if behavioural parameters such as ethograms^9^ or facial grimace scales^10^ are used to assess pain, the approach to pain might vary among veterinarians, as the response to pain is subjective^7^ and depends on the attitude and experience of the observer.^8^

One method to subjectively assess attitudes towards pain in veterinary medicine is by use of pain scales, such as the numerical rating scale (NRS) or visual analogue scale, with the NRS being used most frequently.^11^

Studies assessing the attitudes of veterinarians,^5^, ^12^, ^13^ farmers and producers^14^, ^15^ and other staff^16^, ^17^ towards pain and pain management have been published, with a limited number of studies about the attitudes of veterinary students towards pain in cattle^18^, ^19^ and no published study^20^ about the influence of clinical training in cattle on pain assessment.

The objective of the present paper was to evaluate the influence of 12 weeks of clinical training in a clinic for ruminants on the attitudes of Bavarian veterinary students regarding (i) the estimated severity of pain (pain scores) in cattle and calves for various procedures and diseases; (ii) use of different analgesic regimens; and (iii) statements towards pain in cattle.

## MATERIALS AND METHODS

### Questionnaire

A power analysis for a paired *t*‐test (effect size = 0.5, power = 90%, 20% reserve) indicated that 53 paired surveys were required for statistical evaluation. A ‘moderate’ effect size was used to calculate the number of observations needed in the analysis due to the study being explorative.

A questionnaire with two surveys was developed based on questionnaires used by Huxley and Whay^12^ and Tschoner et al.^14^, ^21^ The questionnaire was developed using the software evasys V9.1 (2467) (evasys, Lüneburg, Germany). Questions were set up as either single‐ or multiple‐choice questions.

Students were asked to complete the questionnaire in their first (Week 1) and last week (Week 12) of clinical training. Participation was voluntary and conducted in accordance with data protection regulations. To pair individual questionnaires from both weeks, students had to create an anonymous individualised code. The questionnaire was accessible online through a link or a QR code, which was provided to students in the first and last weeks of their training during a lecture, as well as via mail. Data were exported via Excel.

A pilot test of the questionnaire was conducted before the start of data collection. Students were asked to test the questionnaire for understandability and conclusiveness. Data collection was conducted from 26 June 2023 to 8 December 2024, and included six different clinical rotation rounds with a total of 93 students.

Both questionnaires contained seven categories:

1. Consent and data privacy protection
2. Code for pairing
3. Demographic data
4. Expression of pain in cattle
5. Pain assessment
6. Necessity of pain management
7. Questions and statements about pain in cattle

The two questionnaires were identical, except for the category assessing demographic data. For the first questionnaire, demographic questions included, among others, questions about age, gender, previous experience with cattle and planned specialisation. For the second questionnaire, demographic questions only inquired about the planned specialisation. For Section 4, students were presented with different behaviours in cattle (e.g., reduced feed intake, bruxism) without images and asked if they considered the individual parameters as signs of pain in cattle. For Section 5, students were asked to evaluate the painfulness of different diseases and procedures in adult cattle and calves under the assumption that no analgesia was provided, using an NRS ranging from 0 (*no pain*) to 10 (*worst pain imaginable*). For Section 6, students had to choose which combination of analgesia they would use for different procedures. The last section consisted of questions about pain in cattle and statement questions. A copy of the questionnaire is given in File S1.

### Clinical training

Students take part in their clinical rotation during the 9th and 10th semesters (out of 11). The rotation is selective according to students’ interests and planned specialisation, and is done at a university‐owned clinic. Students attend the Clinic for Ruminants with Ambulatory and Herd Health Services for 12 weeks, rotating between the four disciplines (Internal Medicine and Surgery, Orthopaedics, Reproduction and Small Ruminants, Ambulatory Services) for 3 weeks each. During this time, they take part in examinations, treatments, rounds, surgeries and other clinical work, including participation in night and weekend shifts.

### Statistical analysis

Data were analysed using R 4.5.0 (2025‐04‐11) statistical software. Categories for Sections 6 and 7 were combined due to a low number of results per category. For Section 6, analgesics were combined as either one analgesic (sedation, local anaesthesia or NSAID), two analgesics (combination of either sedation and local anaesthesia, sedation and NSAID, or local anaesthesia and NSAID) or three analgesics (sedation, local anaesthesia and NSAID). For Section 7, answers were combined as ‘yes’ (yes and rather yes), ‘no’ (no and rather no), and ‘no idea’. A one‐sample proportion test was conducted to compare two proportions between Weeks 1 and 12. McNemar's test was used for paired binary categorical data, while the Cochran *Q* test was employed for paired categorical data with more than two categories. Paired continuous scores (e.g., pain scores) between Weeks 1 and 12 were analysed using the non‐parametric Wilcoxon signed‐rank test. Due to the exploratory design and limited sample size (*n* = 60), unadjusted univariable tests were conducted to maximise sensitivity for potential associations. Given the multiple comparisons typical of questionnaire‐based studies, adjustments such as Bonferroni corrections would drastically reduce statistical power. Thus, we report effect sizes (percentages, medians) alongside *p*‐values, emphasising meaningful associations and acknowledging the elevated risk of false positives. The findings are considered hypothesis generating and require validation in larger independent cohorts. The *p*‐values of 0.05 or less were considered statistically significant.

## RESULTS

### Demographic data

Out of 93 students, 86 (92.5%) answered the first questionnaire, and 65 (69.9%) answered the second one. Of the 65 paired surveys, five surveys had to be excluded from statistical analysis. Reasons for exclusion were: completing the questionnaire after Week 1 of the rotation, which also resulted in the exclusion of the data for Week 12 (*n* = 1), participation after Week 12 of rotation (*n* = 1), participation in only the second survey (*n* = 2) and being unable to pair one questionnaire (*n* = 1). A summary of demographic data is given in Table 1. Planned specialisation did not differ between Week 1 and Week 12 of clinical training.

**TABLE 1:** Demographic data for Bavarian veterinary students (*n* = 60) participating in a survey investigating their attitudes towards pain and pain management in cattle.

Variable	Percentage of respondents (*n* = 60)
**Age**	
20–25 years	65.0%
26–30 years	25.0%
30–35 years	10.0%
>35 years	0%
**Gender**	
Female	93.3%
Male	6.7%
Other	0%
**Where did you grow up?**	
Rural area (<5000 inhabitants)	58.3%
Town (5000–19,999 inhabitants)	16.7%
City (20,000–100,000 inhabitants)	10.0%
Large city (>100,000 inhabitants)	15.0%
**Do you have siblings?**	
None	5.0%
1	38.3%
2	31.7%
3 or more	25.0%
**Did you gain experience with cattle before starting your studies? (multiple answers allowed)**	
No	30.0%
Yes, I grew up on a farm	13.3%
Yes, family/friends own a farm	41.7%
Yes, through work experience	41.7%

	Week 1	Week 12	*p*
**Planned specialisation? (multiple answers allowed)**			
Small animals	38%	42%	0.414
Horses	18%	20%	0.655
Exotic animals	7%	3%	0.517
Cattle/Ruminants	72%	73%	0.782
Food industry	5%	5%	1.000
Pigs	8%	7%	0.317
Poultry	10%	5%	0.083
Not decided yet	32%	22%	0.058

*Note*: The question on planned specialisation was asked in Weeks 1 and 12, and *p*‐values comparing these answers between the two time points were determined by McNemar's test.

### Expression of pain in cattle

The parameter most students relied on for pain assessment was reduced feed intake (95%), followed by an arched back (94%), moaning (94%) and changes in stance or gait (93%) (Table 2). Fever, wrinkling of the skin on the nose and lacrimation were the least frequently chosen pain signals (26%, 24% and 8%, respectively). Recognition of moaning (*p* = 0.046), bruxism (*p* = 0.008), sad, introverted eyes (*p* = 0.008), shivering (*p* = 0.005), wrinkling of skin on the nose (*p* < 0.001) and lacrimation (*p* = 0.014) as signs of pain increased significantly between Weeks 1 and 12.

**TABLE 2:** Parameters chosen by Bavarian veterinary students (*n* = 60) to assess pain in cattle and calves.

Parameter	Week 1 (%)	Week 12 (%)	*p*
Arched back	97	98	0.564
Changes in stance or gait	93	97	0.414
Moaning	93	100	**0.046**
Reduced feed intake	93	93	1.000
Head lowered	90	87	0.414
Difficulties in rising	87	83	0.593
Lameness	87	90	0.527
Rigid, tense posture	87	88	0.763
Tucked up abdomen	87	95	0.096
Increased breathing rate	82	83	0.763
Apathy	80	78	0.796
Bruxism	78	95	**0.008**
Sad, introverted eyes	73	92	**0.008**
Reduced milk yield	65	63	0.808
Increased lying time	63	73	0.157
Ears held back	62	70	0.225
Wide open nostrils	55	58	0.683
Decubitus^a^	53	55	0.827
Rising like a horse	53	67	0.088
Changed social behaviour	48	38	0.134
Bellowing	47	48	0.827
Dull, sunken eyes	45	53	0.297
Emaciation/Cachexia	43	48	0.439
Shivering	38	60	**0.005**
Feed intake while lying	35	35	1.000
Fever	30	25	0.467
Wrinkling of skin on nose	23	48	**<0.001**
Lacrimation	8	18	**0.014**

*Note*: The proportions of students who selected each parameter were compared between Week 1 (before) and Week 12 (at the end) of clinical training via McNemar's test. *p*‐values ≤0.05 were considered statistically significant and are indicated in bold.

^a^ Pressure sore.

### Pain assessment of conditions

In adult cattle, long bone fracture, laminitis, septic arthritis/polyarthritis and uterine torsion were considered the most painful conditions in Week 1 (each with a median score of 8), whereas ketosis (median score 3), chronic mastitis (median score 4) and hypocalcaemia (median score 4) were considered the least painful (Table 3). Pain scoring in Week 12 differed significantly from that in Week 1 for rupture of muscle, decubitus (pressure sore), laminitis, puerperal metritis, uterine prolapse, uterine torsion, left displaced abomasum, intertrigo (dermatitis of skin fold of udder), hypocalcaemia and ketosis (*p* < 0.05).

**TABLE 3:** Median, range and IQR of estimated severity of pain for conditions in adult cattle and calves, as stated by Bavarian veterinary students (*n* = 60) in Weeks 1 and 12 of ruminant clinical rotation, compared via paired Wilcoxon tests.

	Week 1			Week 12			
	Median	Range	IQR	Median	Range	IQR	*p*
**Condition: Adult cattle**							
Fracture of a long bone	8	5–10	7–9	8	4–10	7–9	0.70
Rupture of a muscle	7	3–9	6–9	7	4–10	6.75–9	**0.004**
Intertrigo^a^	5	0–9	4–6	4	0–8	3–6.25	**0.03**
Decubitus^b^	5	1–8	3.5–6	3	1–9	3–5	**0.004**
Laminitis	8	3–10	7–9	7	2–10	6–9	**0.006**
Sole ulcer	6	0–8	4–7	6	1–9	5–7	0.09
Dermatitis digitalis	6	0–9	4–7.25	6	0–10	4–7	0.71
Septic arthritis/polyarthritis	8	3–10	7–9	8	3–10	6.75–9	0.46
Puerperal metritis	7	1–10	6–8	6	1–10	4–8	**0.007**
Uterine eversion	7	0–10	6–8	6	1–10	4–8	**0.002**
Uterine torsion	8	1–10	7–9	7	1–10	5.75–8	**<0.001**
Tissue injuries following birth	6.5	2–10	4–8	6	2–10	5–7.25	0.52
Right displaced abomasum	7	0–10	5–8	6	1–10	5–8	0.66
Left displaced abomasum	7	0–10	5–8	5	1–10	4–7	**0.004**
Chronic mastitis (clots in milk only)	4	0–9	2.75–6	4	1–9	2–5	0.46
Acute mastitis (*Escherichia coli*)	7	0–10	5–8	7	0–10	6–8.25	0.34
Open teat injury	7	3–10	5–8	7	2–10	5–8	0.42
Hypocalcaemia	4	0–8	3–6	3	0–9	2–5	**0.04**
Ketosis	3	0–8	2–6	3	0–9	2–5	**0.04**
Uveitis	6	2–10	4.75–8	7	0–10	5–8	0.43
**Condition: Calves**							
Fracture of a long bone	8	4–10	7–9	8	4–10	6.75–9	0.59
Arthritis/Polyarthritis	8	3–10	8–9	8.5	3–10	7–9	0.70
Contracted tendons	4	0–9	2–6	4	0–10	3–6	0.34
Omphalitis	7	2–10	6–8	7	1–10	5.75–8	0.37
Following dystocia	6	1–10	4–7	6	1–10	4–7	0.47
Ruminal acidosis	6	1–9	4–7	5	0–9	3–7	0.16
Enteritis/Diarrhoea	5	1–8	3.75–7	5	1–10	4–7	0.93
Ileus	8	3–10	7–9	9	2–10	7–10	0.06
Bronchopneumonia	6	0–9	4–7	5	0–10	4–6.25	0.12
Cerebrocortical necrosis	8	0–10	5.75–9	7	1–10	5.75–9	0.53
Meningitis	9	0–10	7–10	8	2–10	7–10	0.57

*Note*: Students were asked to assess pain using a numerical rating scale with the endpoints 0 (*no pain*) and 10 (*worst pain imaginable*), assuming no analgesia was provided. *p*‐values ≤0.05 were considered statistically significant and are indicated in bold.

Abbreviation: IQR, interquartile range.

^a^ Dermatitis of the skin fold of the udder.

^b^ Pressure sore.

In calves, meningitis was considered the most painful condition (median score 9) in Week 1, followed by long bone fracture, arthritis/polyarthritis, ileus and cerebrocortical necrosis (median score of 8 for each) (Table 3). Contracted tendons were considered the least painful condition (median score 4). There were no significant differences in pain scoring between Weeks 1 and 12.

### Pain assessment of procedures

In Week 1 of the rotation, enucleation of the eye bulb (median score 10), claw amputation (median score 9) and caesarean section (median score 9) were considered the most painful procedures in adult cattle, whereas rectal examination and artificial insemination (median score of 1 for each) were considered the least painful (Table 4). Pain scoring was significantly lower in Week 12 than in Week 1 for fetotomy, caesarean section and artificial insemination (*p* < 0.05).

**TABLE 4:** Median, range and IQR of estimated severity of pain for procedures performed in adult cattle and calves, as stated by Bavarian veterinary students (*n* = 60) in Weeks 1 and 12 of ruminant clinical rotation.

	Week 1			Week 12			
	Median	Range	IQR	Median	Range	IQR	*p*‐value
Procedures: Adult cattle							
Treatment of a sole ulcer	7	0–10	5–8	6	0–10	5–8	0.18
Treatment of interdigital hyperplasia	7	2–10	6–8	7	3–10	6–8	0.75
Claw amputation	9	6–10	9–10	10	7–10	9–10	0.24
Dehorning	8	2–10	7–9.25	8	2–10	7–9.25	0.85
Enucleation	10	4–10	9–10	10	5–10	8–10	0.82
Laparoscopic fixation of LDA	7	2–10	6–8	7	2–10	5–8	0.08
Laparotomy	8	2–10	8–9	8	3–10	6–9	0.29
Fixation of the abomasum (Dirksen/omentopexy)	8	2–10	7–9	8	3–10	6.25–9	0.62
Dystocia	8	2–10	7.5–9	8	3–10	6–9	0.21
Fetotomy	7	2–10	5–9	7	0–10	4.75–8	**0.02**
Caesarean section	9	4–10	8–10	8	2–10	7–10	**0.02**
Removal of RFM	5	0–10	3–6.25	4	0–9	2.75–6	0.12
Rectal examination	1	0–10	1–2	1	0–5	0–1	**0.02**
Artificial insemination	1	0–10	1–2	1	0–5	0–2	0.12
**Procedures: Calves**							
Surgical castration	9	3–10	7–10	9	4–10	7–10	0.97
Castration with Burdizzo	9	4–10	7–10	8.5	4–10	7–10	0.24
Umbilical surgery	8	4–10	7–10	9	4–10	7–10	0.06
Laparotomy	9	4–10	8–10	9	2–10	7–10	0.55
Dehorning	8	1–10	6–9	8	1–10	6–9	0.25
Tenotomy of contracted tendons	8	2–10	6–9	8	2–10	6–9	0.95
Intravenous vein catheter placement	2	0–6	1–3	1	0–6	1–2	0.18
Ear tagging	2	0–9	2–4	3	0–9	1–4	0.69

*Note*: Students were asked to assess pain using a numerical rating scale with the endpoints 0 (*no pain*) and 10 (*worst pain imaginable*), assuming no analgesia was provided. *p*‐values ≤0.05 were considered statistically significant and are indicated in bold.

Abbreviations: IQR, interquartile range; LDA, left displaced abomasum; RFM, retained fetal membranes.

In calves, surgical castration, castration with Burdizzo and laparotomy (median score of 9 for each) were considered the most painful procedures, whereas ear tagging and placement of an intravenous ear catheter (median score of 2 for each) were considered the least painful (Table 4). There were no significant differences in pain scoring between Weeks 1 and 12.

### Necessity for pain management

The use of ‘3 analgesics’ was chosen by 87.0%, 80.0% and 75.0% of students for enucleation, claw amputation and laparotomy in cows, respectively, and by 82.0% for both surgical castration and laparotomy in calves (Table 5, Supporting Information S2). No analgesia was deemed necessary for rectal examination (93.0%) and artificial insemination (92.0%) in cows and placement of an intravenous ear catheter (90.0%) and ear tagging (63.0%) in calves. The proportion of agreement was significantly lower for ‘2 analgesics’ for tenotomy of contracted tendons in calves (*p* = 0.003).

**TABLE 5:** Necessity of analgesia for different procedures in adult cattle and calves, as stated by Bavarian veterinary students (*n* = 60).

	1 Analgesic**^a^**			2 Analgesics**^b^**			3 Analgesics**^c^**			None			No idea		
Week	Week 1	Week 12	*p*	Week 1	Week 12	*p*	Week 1	Week 12	*p*	Week 1	Week 12	*p*	Week 1	Week 12	*p*
**Procedures: Adult cattle**															
Treatment of a sole ulcer	30.0	25.0	0.60	45.0	35.0	0.39	15.0	25.0	0.22	8.3	15.0	0.29	1.7	0	0.32
Treatment of interdigital hyperplasia	25.0	15.0	0.22	58.0	52.0	0.62	17.0	30.0	0.13	0	1.7	0.32	0	1.7	0.32
Claw amputation	3.3	5.0	0.65	17.0	15.0	0.82	80.0	80.0	1.00	0	0	NA	0	0	NA
Dehorning	10.0	5.1	0.32	33.0	32.0	0.87	55.0	63.0	0.63	0	0	NA	1.7	0	0.32
Enucleation	3.3	6.7	0.41	10.0	3.3	0.16	87.0	90.0	0.85	0	0	NA	0	0	NA
Laparoscopic fixation of LDA	1.7	6.7	0.18	63.0	43.0	0.17	36.0	47.0	0.32	0	3.3	0.16	0	0	NA
Laparotomy	5.0	5.0	1.00	20.0	33.0	0.16	75.0	62.0	0.38	0	0	NA	0	0	NA
Dirksen/Omentopexy	1.7	8.3	0.10	37.0	32.0	0.64	57.0	60.0	0.81	1.7	0	0.32	3.3	0	0.16
Dystocia	42.0	48.0	0.59	42.0	32.0	0.37	10.0	10.0	1.00	3.3	10.0	0.16	3.3	0	0.16
Fetotomy	27.0	36.0	0.41	37.0	36.0	0.88	30.0	17.0	0.13	5.0	8.5	0.483	1.7	3.4	0.56
Caesarean section	5.0	8.3	0.48	38.0	37.0	0.88	55.0	55.0	1.00	0	0	NA	1.7	0	0.32
Removal of RFM	33.0	33.0	1.00	13.0	5.0	0.13	10.0	3.3	0.16	37.0	53.0	0.17	6.7	5.0	0.71
Rectal examination	5.0	1.7	0.32	0	0	NA	0	1.7	0.32	93.0	97.0	0.85	1.7	0	0.32
Artificial insemination	6.8	3.3	0.41	0	0	NA	0	0	NA	92.0	97.0	0.71	1.7	0	0.32
**Procedures: Calves**															
Surgical castration	5.0	8.3	0.48	13.0	6.7	0.25	82.0	85.0	0.84	0	0	NA	0	0	NA
Castration with Burdizzo	8.3	8.3	1.00	28.0	22.0	0.47	63.0	68.0	0.74	0	0	NA	0	1.7	0.32
Umbilical surgery	3.3	8.3	0.26	15.0	6.7	0.17	80.0	85.0	0.76	0	0	NA	1.7	0	0.32
Laparotomy	3.3	8.3	0.26	15.0	12.0	0.62	82.0	80.0	0.92	0	0	NA	0	0	NA
Dehorning	12.0	8.3	0.56	33.0	17.0	0.07	53.0	75.0	0.14	0	0	NA	1.7	0	0.32
Tenotomy of contracted tendons	8.3	8.3	1.00	37.0	10.0	**0.003**	53.0	80.0	0.07	0	0	NA	1.7	1.7	1.00
Intravenous vein catheter placement	3.3	1.7	0.56	0	0	NA	1.7	1.7	1.00	90.0	95.0	0.78	5.0	1.7	0.32
Ear tagging	25.0	18.0	0.43	1.7	1.7	1.00	1.7	5.0	0.32	63.0	72.0	0.50	8.5	3.3	0.26

*Note*: Results were compared between Week 1 and Week 12 of the ruminant clinical rotation using a one‐sample proportion test. Agreement is given in %. *p*‐values ≤0.05 were considered statistically significant and are indicated in bold.

Abbreviations: LDA, left displaced abomasum; RFM, retained fetal membranes.

^a^ Either sedation, local anaesthesia (LA) or non‐steroidal anti‐inflammatory drug (NSAID).

^b^ Either sedation and LA, sedation and NSAID, or LA and NSAID.

^c^ Combination of sedation, LA and NSAID.

### Questions and statements regarding pain in cattle

A total of 82.0% of students stated that pain recognition in cattle is difficult in Week 1, compared with 67.0% in Week 12 (*p* = 0.039). Overall, 90.0% thought that it is easier to recognise pain in other species (e.g., dogs and horses), both in Weeks 1 and 12 (Table 6, Supporting Information S3). Opinions about farmers’ willingness to pay for analgesia changed significantly (*p* < 0.001) following the clinical training, with 57% answering ‘yes’ in Week 1 and 90% answering ‘yes’ in Week 12. ‘No idea’ was selected by 20% in Week 1 and 0% in Week 12.

**TABLE 6:** Proportion of Bavarian veterinary students (*n* = 60) answering ‘yes’ or ‘no’ to questions asked about pain assessment and management in cattle in Weeks 1 and 12 of a ruminant clinical rotation.

	Yes		No		No idea		
Question	Week 1	Week 12	Week 1	Week 12	Week 1	Week 12	*p*
Is it difficult to recognise pain in cattle?	82.0	67.0	18.0	33.0	0	0	**0.039**
Is it easier to recognise pain in other animals (dogs, horses, …) compared with cattle?	90.0	90.0	8.3	10.0	1.7	0	1.0
Are farm animals less sensitive to pain compared with small and companion animals?	17.0	15.0	78.0	85.0	5.0	0	0.346
Is the owner more capable of recognising pain in their animal than the veterinarian?	37.0	28.0	53.0	57.0	10.0	15.0	0.346
Do animals recover faster from surgery when given analgesia?	85.0	93.0	3.3	1.7	12.0	5.0	0.059
Is a certain level of pain following a surgery helpful, as it decreases movement of the patient?	58.0	55.0	32.0	42.0	10.0	3.3	0.433
Are farmers willing to pay for analgesia to be used in their animals?	57.0	90.0	23.0	10.0	20.0	0	**<0.001**
Do you think that the pain management used in animals treated for diseases at the Clinic for Ruminants is sufficient?	22.0	93.0	1.7	6.7	77.0	0	**<0.001**
Do you think that the pain management used in animals following surgeries at the Clinic for Ruminants is sufficient?	20.0	93.0	1.7	6.7	78.0	0	**<0.001**

*Note*: The differences between Weeks 1 and 2 were analysed using a Cochran test. *p*‐values ≤0.05 were considered statistically significant and are shown in bold. Proportions do not add up to 100% for some questions due to missing answers.

When asked to agree or disagree with given statements (Table 7), 20.0% of students agreed that pain has no protective effect in Week 1, compared with 33.0% in Week 12 (*p* = 0.033). In Week 1, 20.0% of students stated that they had not given any consideration to analgesia in cattle, compared with 2.0% in Week 12 (*p* = 0.003).

**TABLE 7:** Proportion of Bavarian veterinary students agreeing with statements about pain assessment and management in cattle in Weeks 1 and 12 of a ruminant clinical rotation.

Statement	Week 1	Week 12	*p*
Pain has a protective effect as animals do not become too active and recover faster	68.0	60.0	0.251
Pain has no protective effect, as animals recover more slowly when in pain	20.0	33.0	**0.033**
Animals, which are in pain, eat less, which results in lower milk yield and average daily weight gain	93.0	98.0	0.180
Cattle recover faster from diseases/injuries if given analgesic medication	72.0	85.0	0.059
Every pain should be avoided	33.0	37.0	0.564
Minor surgery can be performed without analgesia/anaesthesia to save money and time	13.0	8.0	0.257
Up until now, I paid no attention to the question of analgesia in cattle	20.0	2.0	**0.003**
Treatment costs are not important for farmers; health of their animals and animal well‐being is their top priority	5.0	2.0	0.317
Costs are the main factor that limits farmers from having animals treated with analgesic drugs	53.0	37.0	**0.018**
Farmers rank costs first; costs for treatments for an animal need to stay within certain limits	63.0	47.0	**0.050**
Farmers would be more willing to spend more money on analgesic drugs if the positive effects of analgesic drugs could be perceived economically	72.0	82.0	0.109
Farmers would like their cattle to receive more analgesia, but they are concerned about possible side effects	5.0	17.0	**0.020**
Farmers would like their cattle to receive more analgesia, but they are concerned about withdrawal times	75.0	85.0	0.109

*Note*: The differences between Weeks 1 and 2 were analysed using McNemar's test. *p*‐values ≤0.05 were considered statistically significant and are indicated in bold.

## DISCUSSION

The return rates of 90.3% for Week 1 and 69.9% for Week 12 were higher than for surveys among veterinary students in Norway (57%^18^), Bavarian veterinarians (26.2%^21^), and other European surveys among veterinarians.^2^, ^12^, ^19^, ^22^ Return rates increased after participation was included in the curriculum in the first and last week of the rotation. This may have biased the results, as students could indirectly have felt urged to participate and not have been equally motivated. The distribution of demographic data was similar to Norwegian veterinary students, where 64.3% of students were aged 20–25 years and 80.1% were female,^18^ but differed from previous studies, which included 17.5%–44% of female participants.^2^, ^5^, ^21^, ^23^ However, these studies were conducted among veterinarians and not veterinary students, which could explain the differences in numbers.

The high number of students wanting to specialise in ruminants can be explained by students being able to select their clinical rotation according to their interests, which could have biased the study population towards those particularly interested in farm animals and their health.

Cattle mask their discomfort strongly and only show subtle signs of pain, such as decreased social interaction, flank watching, ear twitching or changed levels of responsiveness.^24^ Therefore, it is easier to recognise obvious behavioural parameters indicative of pain, such as an arched back, changes in stance or gait or reduced feed intake, which were chosen most frequently in our survey. Recognition of subtle changes of behaviour (e.g., lacrimation, pain face) and vocalisation (e.g., bruxism) increased significantly following 12 weeks of clinical training, indicating that veterinary students are not aware that features of the bovine pain face^10^, ^25^ or bruxism^8^ can be used to assess pain before the clinical training. Our results also suggest that time spent working with ruminant health professionals and cattle results in improved recognition of pain expressions, and that training and experience are necessary for assessment of behavioural parameters, which is in accordance with previous research.^8^

Our findings regarding the perceived most painful conditions and procedures are in accordance with data from a previous survey among Bavarian veterinary students^20^ and show similar distributions as described for Norwegian students.^18^ Other surveys also found claw amputation,^5^, ^12^, ^16^, ^21^ caesarean section^2^, ^5^, ^12^, ^21^ and (repair of) fracture of the long bone^5^, ^17^, ^21^ to be assessed with the highest pain scores. However, Bavarian veterinary students rated castration pain higher than practising veterinarians from the UK,^2^, ^12^ Canada^27^ and New Zealand.^5^ Higher awareness of castration pain has also been reported among practising Bavarian veterinarians.^21^ Awareness of pain due to husbandry procedures might be higher in Germany due to the German Animal Welfare Law, given the fact that age restrictions are provided for dehorning, castration and tail docking,^28^ and that dehorning must not be done without sedation and NSAIDs in Cross Compliance‐supported farms.^29^ As 38% and 18% of students participating in this survey wanted to work with either small animals or horses, knowledge about pain management during castration in these species might have influenced our results. Previous studies found that female veterinarians awarded significantly higher pain scores compared with their male colleagues.^2^, ^5^, ^12^, ^21^, ^23^ This was also published for Bavarian veterinary students in 2012.^20^ As only 6.7% (*n* = 4) of our participants were male, we did not assess the difference in pain scores between genders. The high proportion of female participants could be considered a limitation, as previous research indicates that female students tend to express greater empathy towards animals compared to their male counterparts.^30^, ^31^

After 12 weeks of clinical training, mean pain scores were significantly lower for 10 of 20 conditions and three of 15 procedures in adult cattle. This is not in accordance with the results presented in a study from 2012, which stated that students gave significantly higher pain scores for 12 of 23 conditions and for one of 16 procedures, and significantly lower scores for two conditions and three procedures.^20^ The decrease in pain scores for about 50% of diseases in adult cattle could be explained by students gaining experience and possibly seeing these conditions and identifying them as less painful than previously estimated, but it could also be due to changes in the empathy levels of students during their education. Students’ empathy scores are lower in later years of their programmes.^31^ Levels of sentience attributed to various animals by students in their clinical year were found to be lower compared with students in their pre‐clinical year, which indicated that students’ attitudes harden during education to detach themselves from the patients’ suffering.^30^ It is possible that pain scoring changed for conditions and procedures that the students saw, experienced and worked with over the 12‐week clinical training. The practical component of workplace learning tends to have a stronger influence on students’ personal beliefs about animal welfare than academic courses.^31^ We did not assess which conditions and procedures students were familiar with before clinical training, nor did we document which procedures and conditions students experienced during the rotation. As some conditions or procedures are more common than others, we could not guarantee that all students experienced everything, which is a limitation of the study. A person's response to pain is subjective,^7^ depending on their attitude towards and experience with pain.^8^ This was also true for the present study, as the ranges of pain scores given by students show a wide variation, indicating that the perception of pain differs between individual students. As students worked with different veterinarians during the clinical training, their opinions and attitudes could also have rubbed off on students.

For pain management, sedation with xylazine, which is an alpha‐2 agonist, was included as an analgesic due to its mild analgesic effect caused by the inhibition of the release of norepinephrine and substance P.^32^ Chosen analgesic regimens only differed for ‘tenotomy of contracted tendons’, with a significant decrease in ‘2 analgesics’ and an increase in; 3 analgesics;. As the opinion only changed regarding this specific procedure, it is possible that a high number of students experienced this surgery during their rotation. Use of ‘3 analgesics’ decreased about 13% for laparotomy in adult cattle. This could be explained by students often assuming that laparotomies in adult cattle would be performed with animals under general anaesthesia, as in small animals and horses, resulting in lower numbers for sedation following the clinical training. As the rotation was at a university‐owned clinic, with students spending time in speciality hospital services and working with clinicians being teachers, conversations and lectures about treatment options, as well as pain relief, could have influenced the results due to students’ continuing learning on pain management. Clinicians were not blinded to the study, which could further influence the results and bias the study. Students participated in tutorials on claw surgeries and caesarean section in sheep, but not in lectures about pain management to avoid influencing the study's results.

As participation in the study was voluntary, a certain preselection of students interested in pain and pain management in cattle is possible, which might have influenced our results. Additionally, over 50% of students stated that they were interested in specialising in small animals and/or horses. Scales for the assessment of pain have been available for many years for companion animals such as dogs^34^, ^35^ and cats,^36^, ^37^ as well as for horses,^38^, ^39^ raising a higher awareness of pain and pain management in these species. Therefore, different attitudes and opinions of these students, as well as shared lectures on pain management in small and companion animals and horses, could be a reason for our results.

After 12 weeks of clinical experience, agreement with the statement that recognition of pain is difficult in cattle decreased by 15%, indicating that with increasing theoretical and clinical experience, students felt more confident in recognising behaviour associated with pain. However, 67% of students still thought that pain recognition is difficult in cattle, which agrees with previous studies, where veterinarians stated that their knowledge about the recognition and management of pain is not sufficient.^21^, ^23^, ^26^ This indicates that veterinary schools should focus on education on pain management in all species, including cattle. While only 7.7% of Bavarian cattle veterinarians stated that cattle are less sensitive to pain compared with other animals,^14^ 17% and 15% of students agreed with that statement in Weeks 1 and 12, respectively. It is possible that gaining experience as a practising veterinarian over the years changes the perception of pain in cattle. It is alarming that 6.7% of students stated that the pain management at the clinic was inadequate. We did not further explore these statements and, due to the low number of participants, did not assess the influence of demographic data on these results. It is possible that demographic background, as well as planned specialisation (small animals, horses) and experiences during the rotation, could have led to these results.

Due to the low number of participants, we did not assess the influence of demographic data (age, gender, specialisation) on these results, but it is possible that students who planned to specialise in small animals or horses expect or are used to different approaches to pain. We also could not assess the influence of the different clinicians on the students. As students worked with different clinicians and were exposed to their different opinions, this could also have influenced responses to these statements.

Working with farmers positively changed students’ opinions about farmers’ preferences. As veterinary students worked alongside farmers during their rotation and were also included in discussions with farmers about the treatment plan for their animals, their opinions likely changed due to these experiences. Being serviced by a teaching hospital could be a reason why farmers engage in pain management. However, previous research already found that farmers are more willing to pay for analgesia than veterinarians believe them to be.^14^, ^16^, ^22^

As the student population included in this project were from the same institution and received the same training and lectures, the results could be unique to this clinic. Further studies comparing national or international populations should be considered to better assess the effects of training.

According to previous studies, French veterinarians reported that they feel like veterinary school failed to teach them to evaluate pain.^26^ In Germany, 87.6% of Bavarian bovine veterinarians thought that veterinary school did not give them enough information about the assessment of pain in cattle,^21^ and only 59.8% of Brazilian veterinarians stated that they acquired their knowledge about pain assessment in college, with 84.1% of Brazilian veterinarians thinking that their pain recognition skills are not adequate.^23^ Lack of recognition of pain is therefore likely a widespread issue in veterinary medicine.^1^ Inadequate recognition of pain is perceived strongly as a barrier to providing pain management on‐farm.^13^ Therefore, education on pain assessment and recognition, focusing on the expression of pain in cattle, is fundamental to preparing future veterinarians to adequately manage pain in cattle.

## CONCLUSION

The results of this study indicate that clinical training in ruminants influences students’ attitudes and opinions about pain and pain management in cattle, as well as assumptions about farmers’ opinions regarding pain management. This highlights the important role that practical experience plays in veterinary education. Veterinary schools should focus on educating students about how to recognise and manage pain in cattle to improve welfare in these animals.

## AUTHOR CONTRIBUTIONS

**Theresa Tschoner**: Conceptualisation, methodology, formal analysis, investigation, resources, data curation, writing—original draft, visualisation, project administration. **Kristina R. Mueller**: Conceptualisation, resources, data curation, writing—review and editing, visualisation, project administration. **Yury Zablotski**: Methodology, format analysis, data curation, writing—review and editing. **Melanie Feist**: Conceptualisation, formal analysis, investigation, resources, data curation, writing—review and editing, supervision, project administration.

## CONFLICT OF INTEREST STATEMENT

The authors declare no conflicts of interest.

## ETHICS STATEMENT

The study was conducted following approval by the ethics committee of the Department for Veterinary Medicine of the Ludwigs Maximilians University, Munich (reference number 358‐20‐04‐2023).

## Supporting information



Supporting Information

## Data Availability

Data available on request from the authors.

## References

[1] Steagall PV , Bustamante H , Johnson CB , Turner PV . Pain management in farm animals: focus on cattle, sheep and pigs. Animals. 2021;11(6):1483. 10.3390/ani11061483 PMC8223984 34063847

[2] Remnant JG , Tremlett A , Huxley JN , Hudson CD . Clinical attitudes to pain and use of analgesia in cattle—where are we 10 years on? Vet Rec. 2017;181(15):400. 10.1136/vr.104428 28814534

[3] Mich PM , Hellyer PW , Kogan L , Schoenfeld‐Tacher R . Effects of a pilot training program on veterinary students' pain knowledge, attitude, and assessment skills. J Vet Med Educ. 2010;37(4):358–368. 10.3138/jvme.37.4.358 21135403

[4] Cockcroft PD . Pain management in cattle practice. In: Cockcroft PD , editor. Bovine medicine. Wiley Blackwell; 2015. p. 238–245

[5] Laven RA , Huxley JN , Whay HR , Stafford KJ . Results of a survey of attitudes of dairy veterinarians in New Zealand regarding painful procedures and conditions in cattle. New Z Vet J. 2009;57(4):215–20. 10.1080/00480169.2009.36904 19649015

[6] Anil L , Anil SS , Deen J . Pain detection and amelioration in animals on the farm: issues and options. J Appl Anim Welf Sci. 2005;8(4):261–278. 10.1207/s15327604jaws0804_3 16436030

[7] Reader J . Clinicians' attitudes to pain and the use of anagesia in cattle. Vet Rec. 2017;181:397–399. 10.1136/vr.j4724 29030513

[8] Hudson C , Whay H , Huxley J . Recognition and management of pain in cattle. In Pract. 2008;30:126–134.

[9] Feist M , Köstlin R , Nuss K . Claw surgery in cattle: the benefit of perioperative analgesics. Tierarztl Prax G. 2008;36:367–376.

[10] Gleerup KB , Andersen PH , Munksgaard L , Forkman B . Pain evaluation in dairy cattle. Appl Anim Behav Sci. 2015;171:25–32.

[11] Tschoner T , Mueller KR , Zablotski Y , Feist M . Pain assessment in cattle by use of numerical rating and visual analogue scales—a systematic review and meta‐analysis. Animals. 2024;14(2):351. 10.3390/ani14020351 PMC10812761 38275811

[12] Huxley JN , Whay HR . Current attitudes of cattle practitioners to pain and the use of analgesics in cattle. Vet Rec. 2006;159(20):662–8. 10.1136/vr.159.20.662 17099174

[13] Van Dyke R , Connor M , Miele A . An investigation into the perceptions of veterinarians towards perioperative pain management in calves. Animals. 2021;11(7):1882. 10.3390/ani11071882 PMC8300249 34202730

[14] Tschoner T , Sauter‐Louis C , Peinhofer V , Feist M . Exploring the attitudes of Bavarian farmers towards pain in cattle and how they differ from the attitudes of bovine veterinarians. Vet Rec. 2021;189(8):e515. 10.1002/vetr.515 34047373

[15] Wikman I , Hokkanen A‐H , Pastell M , Kauppinen T , Valros A , Hänninen L . Dairy producer attitudes to pain in cattle in relation to disbudding calves. J Dairy Sci. 2013;96(11):6894–6903. 10.3168/jds.2012-6128 24054284

[16] Becker J , Reist M , Friedli K , Strabel D , Wüthrich M , Steiner A . Current attitudes of bovine practitioners, claw‐trimmers and farmers in Switzerland to pain and painful interventions in the feet in dairy cattle. Vet J. 2013;196(3):467–476. 10.1016/j.tvjl.2012.12.021 23369385

[17] Shi R , Shu H , Yu R , Wang Y , Zhang Z , Zhang J , et al. Current attitudes of Chinese dairy practitioners to pain and its management in intensively raised dairy cattle. Animals. 2022;12(22):3140. 10.3390/ani12223140 PMC9686729 36428368

[18] Kielland C , Skjerve E , Zanella AJ . Attitudes of veterinary students to pain in cattle. Vet Rec. 2009;165(9):254–8. 10.1136/vr.165.9.254 19717829

[19] Norring M , Wikman I , Hokkanen A‐H , Kujala MV , Hänninen L . Empathic veterinarians score cattle pain higher. Vet J. 2014;200(1):186–190. 10.1016/j.tvjl.2014.02.005 24685101

[20] Müller KR , Feist M . Attitudes of German veterinary students to pain in cattle and the influence of participation in ruminant clinical rotation on their assessment of pain. Paper presented at the 1st dairy cattle welfare symposium. Guelph, Canada: 2012. p. 212–3.

[21] Tschoner T , Peinhofer VC , Sauter‐Louis C , Feist M . Attitudes of Bavarian bovine veterinarians towards pain and pain management in cattle. Vet Rec. 2020;187:e90. 10.1136/vr.105920 32967935

[22] Thomsen PT , Anneberg I , Herskin MS . Differences in attitudes of farmers and veterinarians towards pain in dairy cows. Vet J. 2012;194(1):94‐7. 10.1016/j.tvjl.2012.02.025 22516921

[23] Lorena SE , Luna SP , Lascelles BDX , Corrente JE Attitude of Brazilian veterinarians in the recognition and treatment of pain in horses and cattle. Vet Anaesth Analg. 2013;40(4):410–8. 10.1111/vaa.12025 23461405

[24] Whay HR , Hudson C , Huxley JN . Where are we with Pain Recognition and Management in Cattle. AABP Proc. 2008;41:54–59.

[25] Feist M , Tschoner T . Ansätze zur Schmerzbeobachtung bei Kälbern–ein Überblick. Vet Spiegel. 2023;33(2):64–73.

[26] Hugonnard M , Leblond A , Keroack S , Cadore JL , Troncy E . Attitudes and concerns of French veterinarians towards pain and analgesia in dogs and cats. Vet Anaesth Analg. 2004;31(3):154–163. 10.1111/j.1467-2987.2004.00175.x 15268686

[27] Hewson CJ , Dohoo IR , Lemke KA , Barkema HW . Canadian veterinarian's use of analgesics in cattle, pigs and horses in 2004 and 2005. CanVet J. 2007;48(2):155–164. 10.4141/cjas68-021 PMC1780232 17334029

[28] Tierschutzgesetz (Animal Welfare Act). Federal Ministry of Justice and Consumer Protection, Germany; 2018. Accessed May 12, 2018. https://www.gesetze‐im‐internet.de/tierschg/BJNR012770972.html

[29] Cross Compliance 2022. BAYERN/DIREKT; 2022. Accessed December 17, 2025. https://www.stmelf.bayern.de/mam/cms01/allgemein/publikationen/broschuere_cross_compliance.pdf

[30] Paul ES , Podberscek AL . Veterinary education and students' attitudes towards animal welfare. Vet Rec. 2000;146(10):269–272. 10.1136/vr.146.10.269 10749039

[31] Pollard‐Williams S , Doyle RE , Freire R . The influence of workplace learning on attitudes toward animal welfare in veterinary students. J Vet Med Educ. 2014;41(3):253–7. 10.3138/jvme.0114-006R1 24981422

[32] Anderson DE , Muir WW . Pain management in cattle. Vet Clin North Am Food Anim Pract. 2005;21:623–635. 10.1016/j.cvfa.2005.07.002 16243627

[33] Anderson DE , Edmondson MA . Prevention and management of surgical pain in cattle. Vet Clin North Am Food Anim Pract. 2013;29:157–184. 10.1016/j.cvfa.2012.11.006 23438404

[34] Holton L , Reid J , Scottt EM , Pawson P , Nolan A . Development of a behaviour‐based scale to measure acute pain in dogs. Vet Rec. 2001;148(17):525–531. 10.1136/vr.148.17.525 11354645

[35] Morton CM , Reid J , Scott EM , Holton LL , Nolan AM . Application of a scaling model to establish and validate an interval level pain scale for assessment of acute pain in dogs. Am J Vet Res. 2005;66(12):2154–2166. 10.2460/ajvr.2005.66.2154 16379662

[36] Brondani JT , Luna SPL , Padovani CR . Refinement and initial validation of a multidimensional composite scale for use in assessing acute postoperative pain in cats. Am J Vet Res. 2011;72(2):174–183. 10.2460/ajvr.72.2.174 21281191

[37] Brondani JT , Mama KR , Luna SP , Wright BD , Niyom S , Ambrosio J , et al. Validation of the English version of the UNESP‐Botucatu multidimensional composite pain scale for assessing postoperative pain in cats. BMC Vet Res. 2013;9:143. 10.1186/1746-6148-9-143 PMC3722032 23867090

[38] Van Loon JP , Back W , Hellebrekers LJ , Van Weeren PR . Application of a composite pain scale to objectively monitor horses with somatic and visceral pain under hospital conditions. J Equine Vet Sci. 2010;30(11):641–9.

[39] Bussières G , Jacques C , Lainay O , Beauchamp G , Leblond A , Cadoré J‐L , et al. Development of a composite orthopaedic pain scale in horses. Res Vet Sci. 2008;85(2):294–306. 10.1016/j.rvsc.2007.10.011 18061637

