# Supplementary material for: Assessing veterinary students’ attitudes towards pain in cattle before and after final‐year clinical training
Source: Vet Rec. 2026 May 13;199(10):e435–44. doi: 10.1002/vetr.70731 (PMC13768725; doi:10.1002/vetr.70731)
Supplement: Supplementary file 1 [file VETR-199--s001.docx]

**Supplement 1:** Copy of the questionnaire given to students to assess their attitudes towards pain and pain management in cattle in week 1 and week 12 of a clinical rotation at the Clinic for Ruminants with Ambulatory and Herd Health Services of the Ludwig-Maximilians-University in Munich, Germany. Both questionnaires were identical, except for demographic data, which, for week 12, only included planned specialisation.

**Supplement 2:** Necessary analgesia for different surgeries in adult cattle and calves as stated by Bavarian veterinary students (n = 60). Results were compared between week 1 (prior to) and week 12 (end of) clinical rotation. Agreement is given in proportion in %.

|  | **Sedation** | | **LA** | | **NSAID** | | **Sedation+LA** | | **Sedation+NSAID** | | **LA+NSAID** | | **Sedation+LA+NSAID** | | **None** | | **No idea** | |
| --- | --- | --- | --- | --- | --- | --- | --- | --- | --- | --- | --- | --- | --- | --- | --- | --- | --- | --- |
| **Surgeries Adult Cattle** | **1** | **12** | **1** | **12** | **1** | **12** | **1** | **12** | **1** | **12** | **1** | **12** | **1** | **12** | **1** | **12** | **1** | **12** |
| Treatment of a sole ulcer | 3.3 | 6.7 | 13 | 5 | 13 | 13 | 5.0 | 6.7 | 5.0 | 6.7 | 35 | 22 | 15 | 25 | 8.3 | 15 | 1.7 | 0 |
| Treatment of an interdigital hyperplasia | 1.7 | 1.7 | 20 | 6.7 | 3.3 | 6.7 | 6.7 | 10 | 5.0 | 5.0 | 47 | 37 | 17 | 30 | 0 | 1.7 | 0 | 1.7 |
| Claw amputation | 1.7 | 5.0 | 1.7 | 0 | 0 | 0 | 1.7 | 1.7 | 0 | 1.7 | 15 | 12 | 80 | 80 | 0 | 0 | 0 | 0 |
| Dehorning^1^ | 0 | 3.4 | 8.3 | 1.7 | 1.7 | 0 | 8.3 | 17 | 15 | 6.8 | 10 | 8.5 | 55 | 63 | 1.7 | 0 | 0 | 0 |
| Extirpation of eye bulb | 3.3 | 5.0 | 0 | 0 | 0 | 1.7 | 1.7 | 1.7 | 3.3 | 0 | 5.0 | 1.7 | 87 | 90 | 0 | 0 | 0 | 0 |
| Laparoscopic fixation of LDA^1^ | 1.7 | 0 | 0 | 6.7 | 0 | 0 | 14 | 8.3 | 22 | 3.3 | 27 | 32 | 36 | 47 | 0 | 0 | 0 | 3.3 |
| Laparotomy | 3.3 | 0 | 1.7 | 5.0 | 0 | 0 | 3.3 | 6.7 | 8.3 | 1.7 | 8.3 | 25 | 75 | 62 | 0 | 0 | 0 | 0 |
| Dirksen/Omentopexie | 1.7 | 0 | 0 | 6.7 | 0 | 1.7 | 6.7 | 5.0 | 13 | 1.7 | 17 | 25 | 57 | 60 | 1.7 | 0 | 3.3 | 0 |
| Dystocia | 3.3 | 0 | 3.3 | 5.0 | 35 | 43 | 3.3 | 1.7 | 5.0 | 8.3 | 33 | 22 | 10 | 10 | 3.3 | 10 | 3.3 | 0 |
| Fetotomy^1^ | 6.7 | 3.4 | 1.7 | 1.7 | 18 | 31 | 3.3 | 3.4 | 13 | 24 | 20 | 8.5 | 30 | 17 | 5.0 | 8.5 | 1.7 | 3.4 |
| Caesarean section | 1.7 | 0 | 1.7 | 5.0 | 1.7 | 3.3 | 5.0 | 3.3 | 5.0 | 0 | 28 | 33 | 55 | 55 | 0 | 0 | 1.7 | 0 |
| Removal of RFM^*^ | 0 | 0 | 6.7 | 0 | 27 | 33 | 1.7 | 0 | 3.3 | 3.3 | 8.3 | 1.7 | 10 | 3.3 | 37 | 53 | 6.7 | 5.0 |
| Local anaesthesia in the flank | 6.7 | 13 | 5.0 | 1.7 | 8.3 | 5.0 | 0 | 1.7 | 1.7 | 3.3 | 0 | 0 | 0 | 3.3 | 68 | 72 | 10 | 0 |
| Rectal examination | 1.7 | 0 | 3.3 | 1.7 | 0 | 0 | 0 | 0 | 0 | 0 | 0 | 0 | 0 | 1.7 | 93 | 97 | 1.7 | 0 |
| Artificial insemination^1^ | 1.7 | 0 | 1.7 | 1.7 | 3.4 | 1.7 | 0 | 0 | 0 | 0 | 0 | 0 | 0 | 0 | 92 | 97 | 1.7 | 0 |
|  | **Sedation** | | **LA** | | **NSAID** | | **Sedation+LA** | | **Sedation+NSAID** | | **LA+NSAID** | | **Sedation+LA+NSAID** | | **None** | | **No idea** | |
| **Surgeries Calves** | **1** | **12** | **1** | **12** | **1** | **12** | **1** | **12** | **1** | **12** | **1** | **12** | **1** | **12** | **1** | **12** | **1** | **12** |
| Surgical castration | 1.7 | 6.7 | 1.7 | 0 | 1.7 | 1.7 | 1.7 | 1.7 | 5.0 | 5.0 | 8.3 | 0 | 80 | 85 | 0 | 0 | 0 | 0 |
| Castration with Burdizzo | 1.7 | 6.7 | 5.0 | 0 | 1.7 | 1.7 | 8.3 | 3.3 | 6.7 | 10 | 13 | 8.3 | 63 | 68 | 0 | 0 | 0 | 1.7 |
| Umbilical surgery | 1.7 | 6.7 | 0 | 0 | 1.7 | 1.7 | 0 | 3.3 | 6.7 | 3.3 | 8.3 | 0 | 80 | 85 | 0 | 0 | 1.7 | 0 |
| Laparotomy | 3.3 | 6.7 | 0 | 0 | 0 | 1.7 | 3.3 | 5.0 | 10 | 3.3 | 1.7 | 3.3 | 82 | 80 | 0 | 0 | 0 | 0 |
| Dehorning | 3.3 | 6.7 | 6.7 | 1.7 | 1.7 | 0 | 10 | 5 | 12 | 5.0 | 12 | 6.7 | 53 | 75 | 1.7 | 0 | 0 | 0 |
| Tenotomy of contracted tendons | 3.3 | 6.7 | 5.0 | 0 | 0 | 1.7 | 12 | 1.7 | 10 | 6.7 | 15 | 1.7 | 53 | 80 | 0 | 0 | 1.7 | 1.7 |
| Intravenous vein catheter placement | 0 | 0 | 1.7 | 1.7 | 1.7 | 0 | 0 | 0 | 0 | 0 | 0 | 0 | 1.7 | 1.7 | 90 | 95 | 5.0 | 1.7 |
| Ear tagging^1^ | 1.7 | 0 | 10 | 1.7 | 14 | 17 | 0 | 1.7 | 0 | 0 | 1.7 | 0 | 1.7 | 5.0 | 63 | 72 | 8.5 | 3.3 |

^1^nA = 1

**Supplement 3:** Agreements with answers to questions regarding pain and pain assessment in cattle as given by Bavarian veterinary students (n = 60) in week 1 and week 12. Results were compared between week 1 (prior to) and week 12 (end of) clinical rotation. Agreement is given in proportion in %.

|  | **Yes** | | **Rather yes** | | **Rather no** | | **No** | | **No idea** | |
| --- | --- | --- | --- | --- | --- | --- | --- | --- | --- | --- |
| **Question** | **1** | **12** | **1** | **12** | **1** | **12** | **1** | **12** | **1** | **12** |
| Is it difficult to recognize pain in cattle? | 8.3 | 8.3 | 73.3 | 58.3 | 16.7 | 28.3 | 1.7 | 5.0 | 0.0 | 0.0 |
| Is it easier to recognize pain in other animals (dogs, horses, …) compared with cattle? | 58.3 | 56.7 | 31.7 | 33.3 | 5.0 | 5.0 | 1.7 | 5.0 | 1.7 | 0.0 |
| Are farm animals less sensitive to pain compared with small and companion animals? | 3.3 | 1.7 | 13.3 | 13.3 | 21.7 | 15.0 | 56.7 | 70.0 | 5.0 | 0.0 |
| Is the owner more capable of recognizing pain in their animal than the veterinarian? | 5.0 | 1.7 | 31.7 | 26.7 | 36.7 | 33.3 | 16.7 | 23.3 | 10.0 | 15.0 |
| Do animals recover faster from surgery when given analgesia? | 51.7 | 51.7 | 33.3 | 41.7 | 3.3 | 1.7 | 0.0 | 0.0 | 11.7 | 5.0 |
| Is a certain level of pain following surgery helpful, as it decreases movement of the patient? | 13.3 | 16.7 | 45.0 | 38.3 | 20.0 | 30.0 | 11.7 | 11.7 | 10.0 | 3.3 |
| Are farmers willing to pay for analgesia to be used in their animals? | 11.7 | 26.7 | 38.3 | 63.3 | 21.7 | 10.0 | 1.7 | 0.0 | 20.0 | 0.0 |
| Do you think that the pain management used in animals treated for diseases at the Clinic for Ruminants with Ambulatory and Herd Health Services is sufficient? | 11.7 | 51.7 | 11.7 | 41.7 | 0.0 | 6.7 | 1.7 | 0.0 | 76.7 | 0.0 |
| Do you think that the pain management used in animals following surgeries at the Clinic for Ruminants with Ambulatory and Herd Health Services is sufficient?^1^ | 11.7 | 63.3 | 8.3 | 30.0 | 0.0 | 6.7 | 1.7 | 0.0 | 76.7 | 0.0 |

^1^nA = 1 in week 1
